# Late Miocene Arctic warmth and terrestrial climate recorded by North Greenland speleothems

**DOI:** 10.1038/s41561-025-01822-0

**Published:** 2025-10-21

**Authors:** Gina E. Moseley, Gabriella Koltai, Jonathan L. Baker, Jian Wang, Heather Stoll, Anika Donner, Lena Friedrich, Christoph Spötl, M. Paul Smith, Denis Scholz, Hai Cheng, Adam Hartland, Clivia Hejny, R. Lawrence Edwards

**Affiliations:** 1 Institute of Geology, University of Innsbruck, Innsbruck, Austria; 2 Institute of Global Environmental Change, Xi’an Jiaotong University, Xi’an, China; 3 Department of Earth Sciences, ETH Zürich, Zurich, Switzerland; 4 Oxford University Museum of Natural History, Oxford, UK; 5 Institute for Geosciences, Johannes Gutenberg University Mainz, Mainz, Germany; 6 Te Aka Mātuatua, School of Science, University of Waikato, Hamilton, New Zealand; 7 Institute of Mineralogy and Petrography, University of Innsbruck, Innsbruck, Austria; 8 School of Earth and Environmental Sciences, University of Minnesota, Minneapolis, MN USA

**Keywords:** Palaeoclimate, Climate change, Cryospheric science, Atmospheric science

## Abstract

The sensitivity of terrestrial Arctic climate during the Late Miocene remains poorly understood, despite this interval marking the transition towards a cooler, more variable global climate and the prelude to Northern Hemisphere glaciation. Here we present a Late Miocene terrestrial proxy record, developed through the analysis of speleothems, from eastern North Greenland (Kalaallit Nunaat). Growth periods indicate multiple episodes of permafrost absence between ~10 and 5 Ma, suggesting mean annual air temperatures ~14 °C higher than present coinciding with atmospheric CO_2_ concentrations above ~310 ppm and local sea surface temperature anomalies >2 °C higher than present. Such moderate thresholds for permafrost absence highlight the climate sensitivity of North Greenland. Spikes in siliciclastic-derived trace elements ~6.3 and ~5.6 Ma are interpreted as terrestrial indicators for Late Miocene ephemeral glaciers in North Greenland. Climate variability recorded during speleothem growth periods was predominantly forced by obliquity, although, in the earliest Late Miocene, obliquity-scale anti-phasing with Antarctica may have occurred. Regional sea-ice extent was at its greatest following ~5.6 Ma during phases of transient glacial–interglacial cycles. Our findings highlight the sensitivity of the Arctic climate system and permafrost to modest CO_2_ levels and provide insights into regional responses to orbital forcing.

## Abstract (web summary)

The Late Miocene terrestrial climate in eastern North Greenland displayed elevated temperatures at moderate atmospheric CO_2_ levels and was highly variable, reflecting the shifting orbital and ocean circulation forcings in the Arctic as the world cooled, according to proxy records from speleothems.

## Main

The Late Miocene (11.63 to 5.33 million years ago (Ma)) was characterized by global warmth relative to preindustrial values^1^, superimposed on a cooling trend that became increasingly pronounced at high latitudes^2^. Reconstructed atmospheric CO_2_ concentrations of ~200–500 ppm (refs. ^3,4^) encompass current values and near-future projections, during a time when global palaeogeography was approaching its modern configuration^5,6^. The Late Miocene therefore provides important insights into climate forcings, feedbacks and variability relevant to long-term outcomes of modern climate scenarios^2^.

Despite its importance for predicting near-future global change^2^, knowledge of Late Miocene Arctic climate variability is limited by scarce terrestrial proxy records. Arctic terrestrial palaeoreconstructions and interpretations rely predominantly on glacial geomorphology^7–11^, marine records^7^^,12–17^ and numerical modelling^9^. Northern Hemisphere high-latitude cooling during the Late Miocene is linked to CO_2_ decline, along with palaeogeographic and vegetation changes^2^^,18^^,19^. However, high-resolution climate records remain scarce, yet they are crucial for validating future climate scenarios and understanding Arctic boundary conditions before Northern Hemisphere glaciations^20^. Greenland is thus a priority for acquiring new proxy data^21^ in order to validate model outputs^21,22^.

This study addresses critical knowledge gaps in terrestrial High Arctic Late Miocene temperature, glacial and permafrost evolution, in addition to orbital forcing of climate and sea-ice variability. Precise, radiometrically dated speleothem growth periods from eastern North (eN.) Greenland provide unambiguous evidence for episodes of permafrost absence between ~10 and 5 Ma. These growth periods align to elevated sea surface temperatures (SSTs) in the northern North Atlantic^5,18,20^ and only moderate atmospheric CO_2_ levels^3,4^. Reproducible multi-proxy time series suggest orbital forcing of temperature and sea-ice extent, alongside terrestrial evidence of Late Miocene glaciation in North Greenland.

## Geological and field setting

**Fig. 1:**
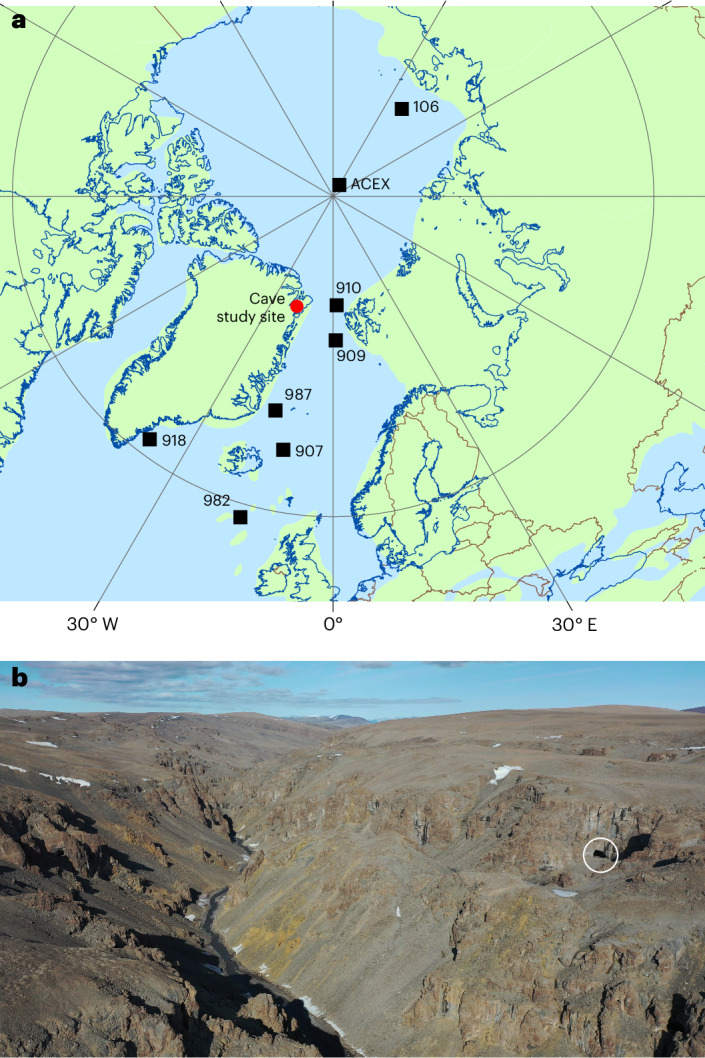
Field location of Cove Cave (Eqik Qaarusussuaq)^23^ relative to other sites discussed in the text. **a**, Present coastlines (blue outline) and country boundaries (brown lines), palaeo-land masses at 8 Ma (ref. ^6^) (green), studied cave location (red circle) and various marine records (black squares). **b**, A photograph of the landscape with the studied cave entrance inside the white circle. Credit: **b**, Robbie Shone. See Extended Data Fig. 1 for further details.

**Extended Data Fig. 1:**
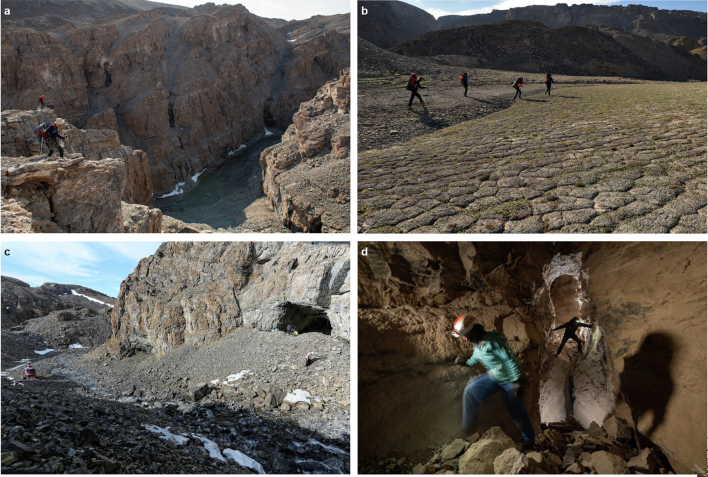
Field location of Cove Cave (Eqik Qaarusussuaq). **a**, Steep-sided canyon with surface river flowing in base. **b**, Thin cryptobiotic soils on the valley floor in Grottedalen displaying patterned ground from the action of permafrost. **c**, Cave entrance with people for scale and helicopter in the valley. This valley is a small hanging tributary valley to (**a**). **d**, Vadose canyon (below figure in background) at the rear of the cave. Flowstone samples were collected from either side of the canyon. See Extended Data Fig. 2 for photo location. Credit: **a**,**b**,**d**, Robbie Shone; **c**, Andrew Sole.

**Extended Data Fig. 2:**
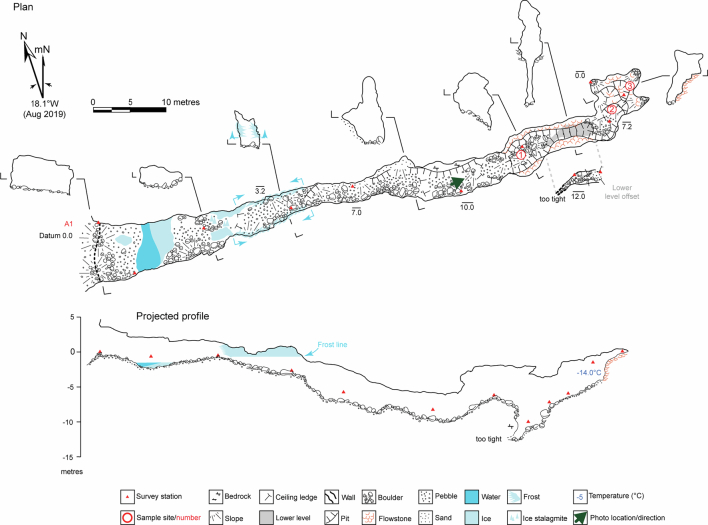
Survey of Cove Cave (Eqik Qaarusussuaq). Adapted from^23^. Samples were collected as follows: 1, KC19-9 and KC19-12. 2, KC19-14. 3, KC19-7. The position of the photo refers to Extended Data Fig. 1d. Figure adapted from ref. ^23^ under a Creative Commons license CC BY.

Four flowstone speleothems were sampled from the rear of Cove Cave (Eqik Qaarusussuaq; 80.3° N, 21.9° W (ref. ^23^); Fig. 1 and Extended Data Figs. 1 and 2) in eN.Greenland, ~35 km west of the coast and ~60 km northeast of the Greenland ice sheet (Fig. 1). Currently, the plateau above the cave lacks an ice cap, although one existed in 1983. In 1889, rapid surface melting caused water to enter the cave, freeze and form cryogenic cave minerals^24^, as occurs when cave air temperature remains below freezing (that is, in permafrost).

The cave, located ~660 m above sea level (asl) and ~30 m below the plateau surface, lies within a tributary valley connected to a steep-sided canyon (Extended Data Fig. 1). Quaternary fluvial incision truncated the cave, leaving it shorter than during speleothem growth.

**Extended Data Fig. 3:**
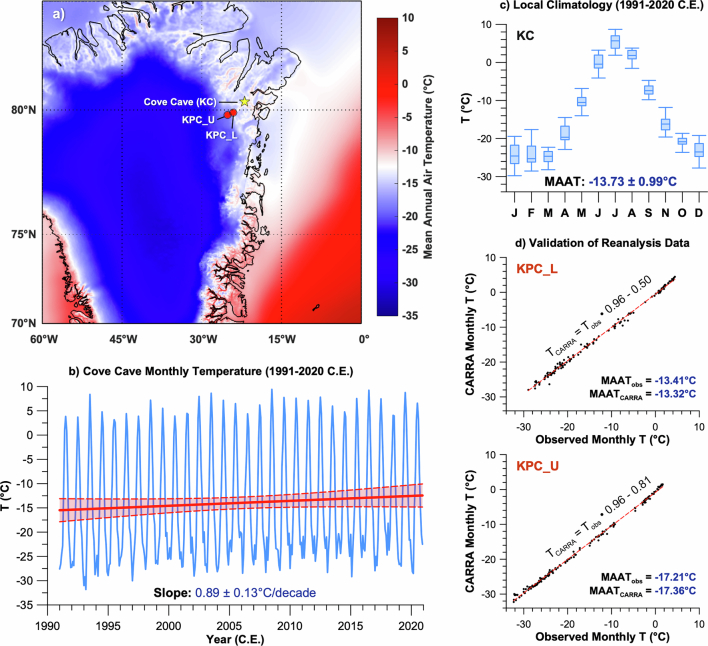
Mean 2 m air temperature at the field site in eastern North Greenland (Kalaallit Nunaat). **a**, Mean 2 m air temperature 1991-2020 as reconstructed from the Copernicus Arctic Regional Reanalysis dataset^57^. The cave site is marked KC. Weather stations of the Programme for Monitoring of the Greenland Ice Sheet & Greenland Climate Network^79^ are marked KPC_L and KPC_U. **b**, Monthly surface temperature at the cave site in the Copernicus Arctic Regional Reanalysis dataset. **c**, Recent climatology (1991–2020) for the cave site and estimated mean annual air temperature. Box and whisker plots for individual months (n = 30). Boxes indicate median and interquartile range (25-75th percentile); whiskers denote 5-95th percentile range. **d**, Reanalysis data in the Copernicus Arctic Regional Reanalysis dataset validated from nearest weather stations.

Modern arid conditions (~200 mm per year (ref. ^25^) and continuous permafrost^26^ limit water infiltration into the cave, therefore preventing vadose speleothem formation. Patterned ground and cryptobiotic soils are scarce on the plateaus but present in valley bottoms (Extended Data Fig. 1). The mean annual air temperature (MAAT; 1991–2020) of −13.7 ± 1.0 °C at the cave location is consistent with spot cave air measurements of −14 °C recorded at the sampling site^23^ (Extended Data Fig. 2). Only 3 months per annum were above freezing, and a warming trend of 0.9 ± 0.1 °C per decade has been recorded (Extended Data Fig. 3).

## Radiometrically dated speleothem growth

**Extended Data Fig. 4:**
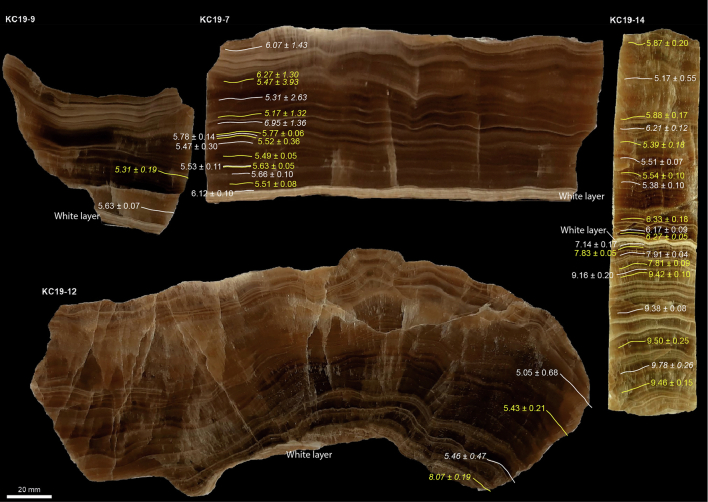
Cut and polished speleothem samples from Cove Cave (Eqik Qaarusussuaq) analysed in this study. including KC19-9, KC-19-7, KC19-14 drill core, KC19-12. U-Pb ages and their positions are shown. White and yellow text is for ease of viewing. Italicised ages are not included in the final age model.

**Extended Data Fig. 5:**
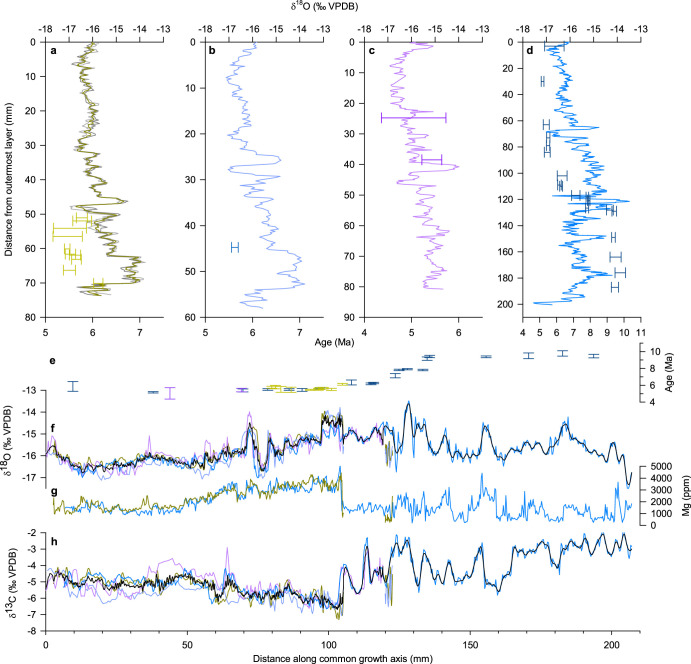
Reproducibility of records and construction of the common depth scale. **a**, KC19-7 δ^18^O isotope track 1 (light grey); KC19-7 δ^18^O isotope track 2 (dark grey); KC19-7 δ^18^O composite record of KC19-7 tracks 1 and 2 (dark yellow). U-Pb ages plotted as mean and propagated 2 standard error (light yellow bars with caps). Individual statistics given in Supplementary Table 1. **b**, KC19-9 δ^18^O (light blue). U-Pb ages plotted as mean and propagated 2 standard error (medium blue bars with caps). Individual statistics given in Supplementary Table 1. **c**, KC19-12 δ^18^O (light purple). U-Pb ages plotted as mean and propagated 2 standard error (medium purple bars with caps). Individual statistics given in Supplementary Table 1. **d**, KC19-14 δ^18^O. U-Pb ages plotted as mean and propagated 2 standard error (dark blue bars with caps). Individual statistics given in Supplementary Table 1. **e**, All ages with a 2-sigma uncertainty range given on the common depth scale. Colours same as for **a**-**d**. **f**, δ^18^O records on the common depth scale. KC19-7_composite_ (yellow); KC19-9 (light blue); KC19-12 (light purple); KC19-14 (medium blue). Composite stacked δ^18^O record (black). **g**, Mg concentration of KC19-7 (yellow) and KC19-14 (medium blue). **h**, As for **e** but for δ^13^C.

The speleothems (code KC) consist primarily of brown calcite, except for a distinctive, thin white layer that is macroscopically, microscopically and geochemically consistent across all samples (Extended Data Fig. 4). Stable-isotope profiles of δ^18^O and δ^13^C are reproducible throughout the entire common record, enabling a stacked composite record (Extended Data Fig. 5). The only exception is the base of KC19-14, which extends further back in time. Given the strong reproducibility in the younger section of the record, we also regard the older part of KC19-14 as reliable, although not yet replicated.

**Fig. 2:**
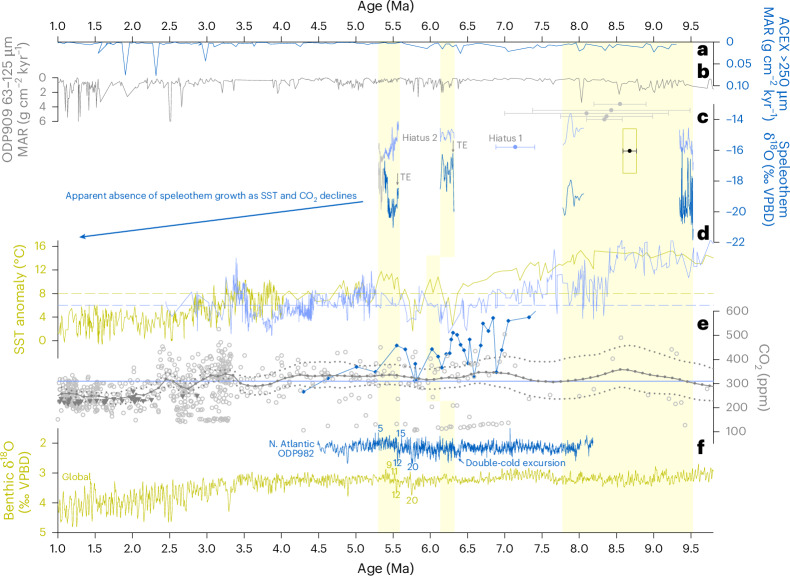
Eastern North Greenland speleothem time series compared with other proxies over the interval 1–9.8 Ma. **a**, Mean accumulation rate (MAR) of >250-μm fraction at the central Arctic ACEX site^15^. **b**, MAR of 63–125-μm fraction at ODP Site 909 in the Fram Strait^17,33^. **c**, U–Pb ages for other caves discussed in the text (grey circles), with uncertainties shown as means and propagated 2 standard error bars. Individual statistics given in Supplementary Table 1). Eastern North Greenland original speleothem δ^18^O composite curve (light blue, this study) and prior calcite precipitation (PCP)-corrected speleothem δ^18^O composite curve (medium blue, this study). Less reliable age model (grey, this study) and singular age (light-blue circle, 2*σ*; Supplementary Discussion 2). Siberian speleothem age and uncertainty (black circle, 2*σ*) surrounded by its δ^18^O range (open yellow box)^27^. TE indicates the position of trace element spikes. **d**, Sea surface temperature (SST) anomalies (U^k^^′^_37_) relative to modern at ODP Site 982 (yellow) and ODP Site 907 (blue)^18^. Horizontal dashed lines highlight thresholds above which speleothem deposition occurred. **e**, Community-vetted atmospheric CO_2_ records (circles) with 100-kyr mean statistical reconstructions shown as median (grey solid) and 95% credible intervals (grey dotted)^3,4^. Antarctic ice CO_2_ (grey triangles)^35^ and high-resolution S. Atlantic *p*CO_2_ record (blue circles)^36^. CO_2_ threshold above which speleothem growth occurred (blue horizontal line). **f**, Benthic δ^18^O ODP Site 982^37,39^ (blue) and global stack^49^ (yellow). Numbers refer to marine oxygen isotope stratigraphy^40^. Vertical yellow bars highlight speleothem growth periods.

**Extended Data Fig. 6:**
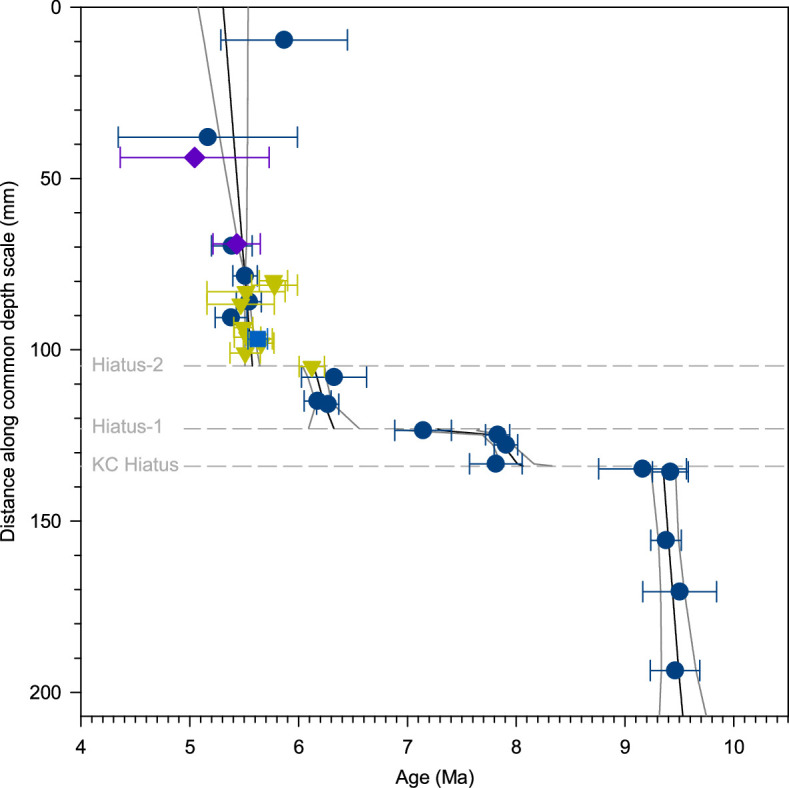
The age model for the composite stacked record constructed in OxCal^66^^,67^. KC19-7 (yellow triangles), KC19-9 (light blue squares), KC19-12 (purple diamonds), KC19-14 (medium blue circles). U-Pb ages plotted as mean and propagated 2 standard error. Individual statistics given in Supplementary Table 1. The solid black line indicates the central age model. Solid grey lines indicate 2-sigma uncertainty on the age model. Horizontal grey dashed lines indicate the position of hiatuses.

U-series (U–Th and U–Pb) dating and Bayesian age modelling of the KC samples indicate four depositional phases: ~9.5–9.3, ~8.1–7.8, ~6.3–6.1 and ~5.6–5.3 Ma, with a possible brief interval ~7.2 Ma (Fig. 2, Extended Data Fig. 6, Supplementary Discussions 1 and 2 and Supplementary Table 1). The mean uncertainty across the age model is 0.8% (standard deviation 0.5, range 0.01–2.6%). However, preliminary U–Pb ages from another cave in the region (15 km northeast) indicate that deposition may have continued between 9.3 and 8.1 Ma, indicating this hiatus is not widespread (Fig. 2). Moreover, speleothem growth in the Siberian Arctic 2,000 km to the south^27^ (72° N; Fig. 2) occurred 8.68 ± 0.09 Ma. Given the preliminary ages from our other field site, we do not continue with a detailed proxy interpretation for this particular sample, but consider growth to have been more or less continuous between ~9.5 and 7.8 Ma (Fig. 2). Hiatuses 1 and 2 occurred between ~7.8 and 6.3 and ~6.1 and 5.6 Ma, respectively, with final growth ending at ~5.3 Ma near the Miocene–Pliocene boundary (Fig. 2). We rule out Quaternary growth at this site (Supplementary Discussion 2), but acknowledge possible short-lived episodes in eN.Greenland based on evidence from other Northern Hemisphere high-latitude sites^28–30^.

## Climatic drivers of speleothem growth

**Extended Data Fig. 7:**
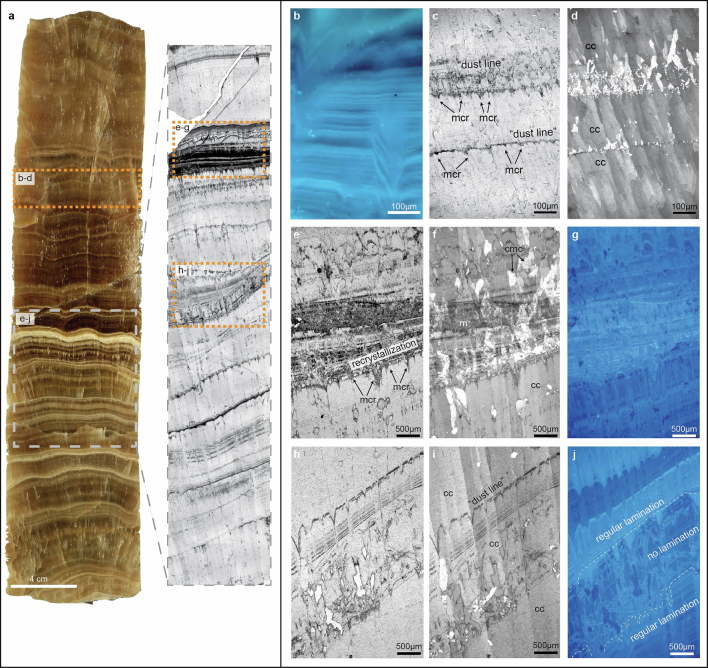
Thin section photomicrographs of KC19-14. **a**, Main slab of KC19-14 and a panoramic view of a thin section showing the areas of photomicrographs **b**-**d** and **e**-**j** (orange dashed rectangles), respectively. **b**, Semi-regular fluorescent lamination seen under UV. **c**, Thin “dust lines” rich in opaque inclusions mark possible growth hiatuses in plane-polarised light. **d**, The same section under cross-polarised light, showing laterally discontinuous interruption of columnar crystal growth. **e** to **g** are thin section images of hiatus-1 (as defined in main text, see Extended Data Fig. 6), showing a sharp transition from columnar calcite below to micritic calcite above. **h** to **j** are thin section images of ‘KC-hiatus’ (as described in main text, see Extended Data Fig. 6), showing columnar calcite fabric. Ultraviolet fluorescence reveals a ca. 1.5 mm-thin zone lacking regular fluorescent lamination. The micritic zone shows brighter epifluorescence than the columnar calcite. cc: columnar calcite, cmc: columnar microcrystalline calcite, m: micrite, mcr: micro-corrosion.

Speleothem deposition during the Late Miocene at ~9.5–7.8, ~6.3–6.1 and ~5.6–5.3 Ma indicates a more humid climate with ground and cave-air temperatures above 0 °C, implying the absence of permafrost. Regular fluorescent lamination (Extended Data Fig. 7) indicates soil and vegetation were above the cave throughout all depositional phases^31^, whereas speleothem δ^13^C values below those of the local limestone bedrock (−0.1 ± 0.1‰; Extended Data Fig. 5) further exclude growth in a subglacial environment or barren snowscape^31^. While higher Late Miocene precipitation^32^ could have enhanced winter ground insulation (Supplementary Discussion 3), this effect is likely to be offset by a longer warm season. We therefore suggest that the MAAT was above freezing during speleothem growth phases, indicating it was at least 13.7 ± 1.0 °C higher than the 1991–2020 mean.

Comparison of the speleothem growth phases with SST anomalies indicates that deposition occurred when SSTs exceeded modern values by >8 °C in the northern North Atlantic (ODP Site 982)^5,18^ and >6 °C in the Iceland Sea (ODP Site 907)^18,20^ (Fig. 2). Considering a persistent ~5 °C SST offset between ODP Site 907 and the central Arctic (ACEX) during the Late Miocene^20^, similar to today^18^, SSTs in the Arctic Gateway near our field site were probably ~3–4 °C cooler than at ODP Site 907^18^. Accordingly, based on current understanding of North Atlantic SST knowledge and chronologies, speleothem deposition probably occurred when local SST anomalies in the Arctic Gateway exceeded 2–3 °C above modern values. Interestingly, SSTs during hiatus 1 also occasionally exceeded this threshold. While this could reflect sampling bias, a major reorganization of ocean circulation in the Arctic Gateway around ~7.5 Ma, related partly to strengthening of the Atlantic Meridional Overturning Circulation and the North Atlantic Current^33^, may have triggered regional climate changes that led to a cessation of speleothem growth.

Critically, community-vetted atmospheric CO_2_ estimates^3,4^ indicate that periods of eN.Greenland speleothem growth occurred at CO_2_ levels above 310 +96/−73 ppm, thereby supporting elevated temperatures at only moderate CO_2_ concentrations^34^. Notably, periods of growth cessation also occurred above this CO_2_ concentration (Fig. 2). However, large scatter, limited variability and sparse early Late Miocene CO_2_ data hinder a thorough assessment of speleothem growth in relation to CO_2_ forcing. Evidence from Pliocene-age Antarctic ice^35^ now confirms that past CO_2_ levels were on the lower end of marine-based reconstructions (Fig. 2). This suggests that hiatuses in speleothem growth may have coincided with periods of reduced CO_2_ concentrations. This is supported by a high-resolution *p*CO_2_ reconstruction from the South Atlantic that spans the Late Miocene Cooling^36^. Although this record generally shows elevated CO_2_ concentrations compared with other records, high-frequency CO_2_ variability is observed across the Late Miocene Cooling with distinctive drops in CO_2_ during both hiatuses 1 and 2 (Fig. 2).

Regardless of the CO_2_ record that is chosen, modern atmospheric CO_2_ concentrations have now surpassed thresholds that in the Late Miocene enabled speleothem growth. Over recent years (1991–2020), eN.Greenland has warmed at 0.9 ± 0.1 °C per decade. Sea ice in the Arctic gateway is also at a record low, thereby leading to reduced ice–albedo feedbacks that are important for permafrost stability^29^. It is therefore possible that the region has already surpassed a tipping point. If warming continues to an equilibrium state where sea surface temperatures in the Arctic Gateway reach 2–3 °C above modern levels, evidence from the speleothem record and current warming rates suggest that complete permafrost thaw in the upper tens of metres could occur within the next one to two centuries. This is in line with predictions for summer sea-ice loss in the Arctic Ocean^20^.

## Late Miocene glacial expansion

The cessation of speleothem deposition during hiatuses 1 and 2 (Fig. 2) may be attributed to preservation or sampling biases; however, the timing of these hiatuses suggests that regional cooling and/or aridification probably inhibited speleothem growth. Marine records from these intervals indicate substantial changes in both surface^5,18,20^ and deep ocean^33^ conditions (Fig. 2). Notably, North Atlantic SSTs declined during the Late Miocene^5,18^ with enhanced high-latitude cooling during hiatus 1 (ref. ^5^). Furthermore, two major SST collapses^18^ occurred during hiatuses 1 and 2, falling below temperature thresholds required for speleothem formation (Fig. 2). After the final growth cessation, SST and CO_2_ levels remained consistently below these thresholds throughout much of the Pliocene and Quaternary. This prolonged cooling trend likely promoted permafrost development, explaining the absence of speleothem deposition during these later intervals (Fig. 2).

Although there is a slight offset between the ~6.3–6.1 Ma speleothem growth phase and the peak in regional SST, the pattern and duration of SST variability are broadly consistent with speleothem growth, suggesting the offset may be due to chronological uncertainty (Fig. 2). As SSTs declined during the growth hiatuses, eN.Greenland MAAT likely dropped as well, potentially promoting permafrost aggradation and halting speleothem formation. Northern North Atlantic benthic δ¹⁸O (ODP Site 982) records a double cold excursion (~0.7‰) between ~6.4 and 6.3 Ma (ref. ^37^), coinciding with the first SST collapse^18^ and the end of hiatus 1. Hiatus 2 aligns with extensive Antarctic ice expansion^38^ as well as a series of transient glacial–interglacial cycles in which the most prominent was TG20^39,40^.

**Extended Data Fig. 8:**
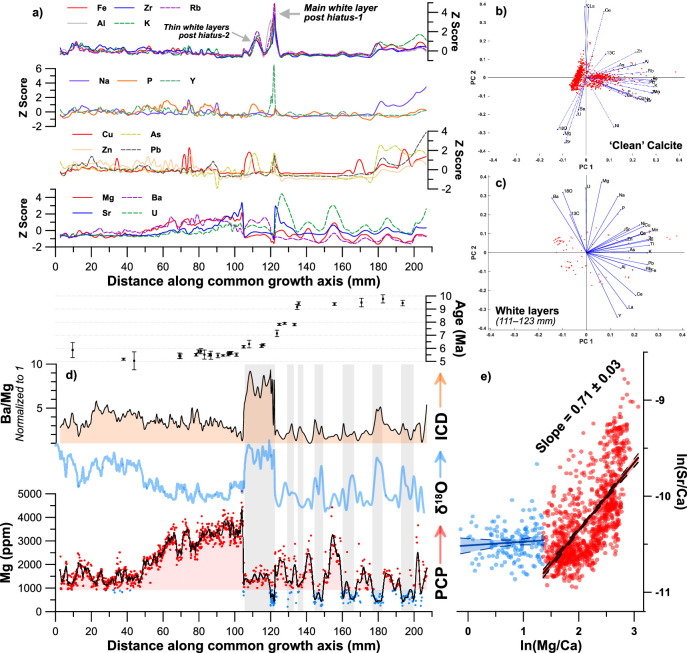
Major and trace elements of KC19-7 and KC19-14. **a**, Selected elements associated with silicate weathering (Fe, Zr, Rb, Al, K), sea spray (Na) and colloidal soil transport (P, Y), sulphide oxidation (Cu, As, Zn, Pb), and prior calcite precipitation (PCP; Mg, Sr, Ba, U). Data standardised to Z-scores for comparison. Z scores calculated as standard deviation units of each time series (n = 1179), Z = (xi – μ / σ). **b**, Principal component 1 and 2 scores for the bulk calcite (excluding detrital-rich white layer). **c**, Principal component 1 and 2 scores for detrital-rich white layer. **d**, U-Pb ages (2-sigma uncertainty) on common depth scale plus proxies for enhanced incongruent calcite dissolution (Ba/Mg, normalised to the lowest value) and for prior calcite precipitation (Mg; values above/below ~920 ppm in red/blue). Grey bars show intervals of enhanced incongruent calcite dissoluation and reduced prior calcite precipitation during warm climates, inferred from prior calcite precipitation-corrected δ^18^O. **e**, ‘Sinclair Test’ for prior calcite precipitation enrichment of Mg and Sr, positive only when Mg exceeds ~920 ppm.

**Extended Data Fig. 9:**
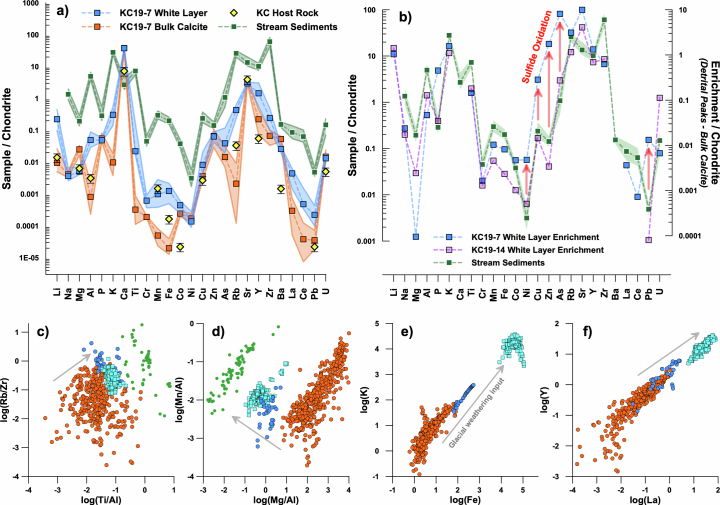
Glacial provenance of white layer trace-element enrichment. **a**, Spider diagram of C1 chondrite-normalised^80^ elemental concentrations of the brown calcite (orange) and distinct white layer (blue) in sample KC19-7, compared to the carbonate host rock (yellow) and a compilation of modern, glacially discharged stream sediment samples^81^ (green) within ~100 km of KC Cave (Eqik Qaarusussuaq). Bedrock values (yellow diamonds) plotted as the arithmetic mean of triplicate measurements, error bars show ± 30%. Dashed lines and shading indicate median and interquartile range (25–75th percentile) of KC19-7 bulk calcite (n = 502), white layer (n = 35), and stream sediments (n = 300). **b**, Spider diagram showing the composition of trace-element enrichment within the distinct white layer of KC19-7 (blue) and KC19-14 (pink), compared to the composition of stream sediments. To quantify trace-element enrichment, we applied the same normalisation to the difference (in ppm) between white layer peaks and bulk calcite values. Red arrows highlight the enhanced enrichment of sulphide-associated metals. Shading in a and b shows the interquartile range of respective datasets. **c, d, e, f**, cross plots of common proxies for detrital provenance in a log-log space, illustrating mixing between local carbonate host rock (captured by the brown calcite, orange circles) and glacially discharged stream sediments (light blue squares) to produce the composition of the white layer (blue circles). Green dots in **c** and **d** show the composition of igneous emplacements and siliciclastic sequences within the Ellesmerian and Caledonian deformation belts of North and East Greenland^82–84^, far more distal from KC Cave (Eqik Qaarusussuaq).

Deep-ocean records from the nearby Fram Strait (ODP Site 909) show ice-rafted debris^17,33^ (IRD) during hiatus 2, while ACEX^15^ contains IRD during both hiatuses (Fig. 2). Quartz grains with glacial microtextures are also found at ACEX around ~7.0, ~6.0 and ~5.5 Ma (ref. ^41^). Farther south, dropstones off central East Greenland (ODP Site 987) indicate IRD deposition began ~7.5 Ma (ref. ^14^), with episodic IRD from central East Greenland reaching offshore South-East Greenland (ODP Site 918) by ~7 Ma (ref. ^13^). There is thus substantial evidence for ocean-terminating glaciers on Greenland during hiatuses 1 and 2. IRD provenance^13^, geomorphological and modelling studies^8,9,42^ all suggest that Late Miocene glaciation was confined to the higher elevations of South-East, southern East, and central Greenland. However, following hiatus 1, all speleothem samples exhibit a distinct white calcitic layer (Fig. 2 and Extended Data Fig. 4) marked by abrupt increases in siliciclastic-derived trace elements (Extended Data Fig. 8). A smaller spike of the same composition is also present following hiatus 2. The elemental signature falls between local carbonate bedrock and recently eroded silicate material from glacial outwash sites (Extended Data Fig. 9) on Precambrian sequences of the former Caledonian highlands (~10 km away). The trace element spikes following hiatuses 1 and 2 are therefore considered to capture the chemical imprint of ice retreat. While the full extent of these glaciations remains uncertain, this represents a terrestrial indication of Late Miocene glacial expansion in eN.Greenland. Our speleothem record supports modelling studies suggesting substantial glacial expansion also occurred in North Greenland during the Late Miocene^11^. In the model, ice responded sensitively to temperature variability but formed rapidly under cold conditions^11^; we therefore suspect a glaciation occurred near the end of hiatus 1, probably at the time of the North Atlantic ‘double cold excursion’^37^, the first distinctive drop in SST^18^ and IRD at the ACEX site^15^.

## Orbital forcing, sea-ice extent and hemispheric phasing

**Fig. 3:**
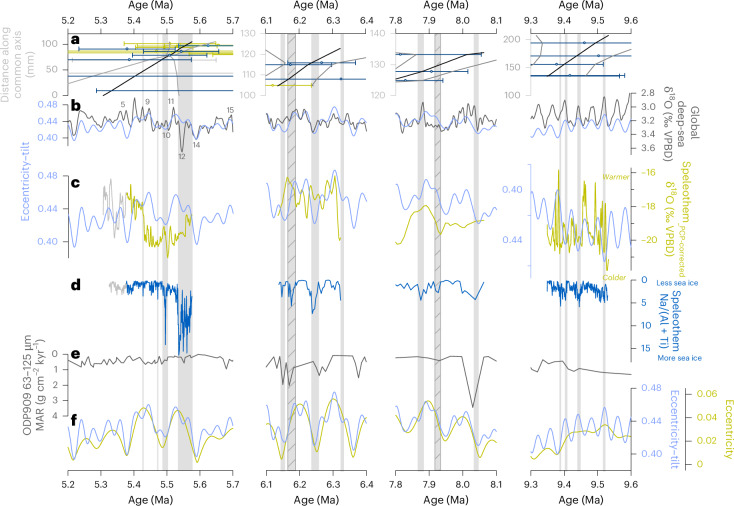
Time-series of periods of eastern North Greenland speleothem deposition. **a**, U–Pb ages (horizontal bars are uncertainties plotted as mean and propagated 2 standard error uncertainty. Individual statistics given in Supplementary Table 1) and Bayesian age model (centre age in black, 95% confidence in grey) as described in text. KC19-7 (yellow), KC19-9 (medium blue), KC19-12 (grey), KC19-14 (dark blue). **b**, Global benthic δ^18^O stack^49^ (grey). Numbers highlight ‘TG’ marine isotope stratigraphy^40^. Eccentricity–tilt composite^47,48^ (blue). **c**, Eastern North Greenland PCP-corrected δ^18^O composite curve (yellow). Less reliable age model (grey) (Supplementary Discussion 2; this study). Eccentricity–tilt composite^47,48^ (blue). **d**, Eastern North Greenland Na/(Al + Ti) record (blue; this study). Less reliable age model (grey) (Supplementary Discussion 2). **e**, Mean accumulation rate (MAR) of 63–125-μm fraction at ODP Site 909 in the Fram Strait^17,33^. **f**, Orbital eccentricity^48^ (yellow) and eccentricity–tilt composite^47,48^ (blue). Vertical grey bars highlight sea-ice expansion events. Solid bars align with obliquity nodes, while hatched bars indicate sea-ice expansion events associated with alternative orbital forcing.

**Extended Data Fig. 10:**
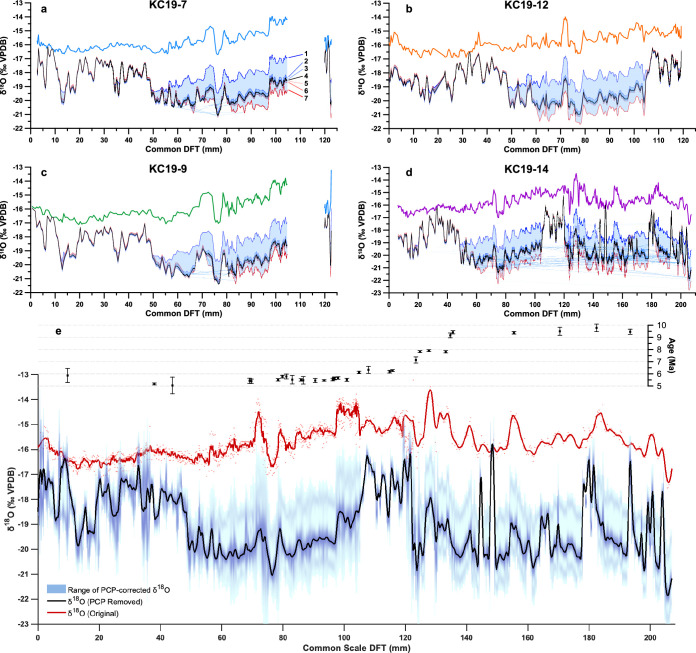
Removal of prior calcite precipitation effect from measured δ^18^O. Measured δ^18^O in **a**, KC19-7 (blue), **b**, KC19-9 (green), **c**, KC19-12 (orange), and **d**, KC19-14 (purple), corrected for the ^18^O-enrichment from prior calcite precipitation^73^. Parameters for pCO_2_, temperature (T), and water film thickness (D) were varied in 7 scenarios to conduct a sensitivity test of the model: 1 - pCO_2_ = 1500 ppm; T = 1 °C; D = 0.015 mm; 2 - pCO_2_ = 3550 ppm; T = 1 °C; D = 0.01 mm; 3 - pCO_2_ = 4370 ppm; T = 4 °C; D = 0.01 mm; 4 - pCO_2_ = 5000 ppm; T = 6 °C; D = 0.01 mm; 5 - pCO_2_ = 5750 ppm; T = 8 °C; D = 0.01 mm; 6 - pCO_2_ = 7080 ppm; T = 11 °C; D = 0.01 mm; 7 - pCO_2_ = 15000 ppm; T = 15 °C; D = 0.005 mm. **e**, U-Pb ages plotted as mean and propagated 2 standard error on common depth scale. Individual statistics given in Supplementary Table 1. Composite of measured δ^18^O data for all KC samples (red). Normalised kernel density function (blue shading) of prior calcite precipitation-corrected δ^18^O, estimated from all model outputs with parameters in scenarios 1-7 and using a 0.6-mm window to account for unequal sampling resolutions. The bold black line shows the maximal probability at each step. DFT = distance from top.

Speleothem δ^18^O time series have been corrected to account for the maximum potential δ^18^O enrichment from prior calcite precipitation (PCP)^31^ (Extended Data Fig. 10; Supplementary Discussion 4). The original (climate-driven) δ^18^O signal of groundwater bicarbonate therefore lies between the measured and PCP-corrected extremes. Regardless of the absolute values, the resulting variability appears robust across a range of model parameters, and the PCP-corrected record restores higher-amplitude δ^18^O shifts and oscillations muted in the original data. This variability forms the basis of the discussion, and we interpret the trends as reliable indicators of isotopic variability driven by regional climate change (Fig. 3). Because regional uplift ceased before speleothem deposition^11,43^, elevation changes are unlikely to have influenced the δ¹⁸O signal. In addition, as used in polar ice core studies^44^, Na was analysed as a proxy for the sea-salt component of marine aerosols (Supplementary Discussion 5). On Greenland’s east coast, Na primarily derives from sea-ice surfaces^45,46^, with elevated concentrations indicating greater sea-ice extent^45^. To isolate the marine aerosol signal (Na_ss_), Na was normalized to the siliciclastic elements Al and Ti, accounting for minor mineral dust contributions^46^ (Fig. 3). The fluctuating sea-ice cover recorded in the speleothems thus aligns with Late Miocene Arctic Ocean biomarkers, which indicate irregular spring sea ice and ice-free summers^20^.

The following discussion into the timing of climatic variability utilizes the independent, radiometrically dated age-modelled chronology. Nonetheless, we acknowledge the age uncertainties and recognize that further research is needed to draw more definitive conclusions.

As noted elsewhere, obliquity forcing played a dominant role in Late Miocene climate variability^2,37,47^. While speleothem δ^18^O variability during all growth phases generally reflects orbital pacing, we observe that the relationship is especially pronounced during the low-amplitude eccentricity cycles between ~9.6 and 9.3 Ma (Fig. 3), consistent with previous observations^47^. On this particular chronology, however, the relationship between δ^18^O and orbital forcing appears inverted when compared with other growth periods. Specifically, higher δ^18^O values (warmer temperatures), correlate with low eccentricity–tilt^47,48^ and increased benthic δ^18^O indicating greater ice volume. Na_ss_ is generally low throughout the growth phase, but increases occasionally with temperature declines. The orbitally tuned global benthic δ^18^O record^49^ naturally shows a clear relationship with eccentricity–tilt across all phases (Fig. 3). However, between ~9.6 and 9.3 Ma, the ice volume signal in the benthic δ^18^O record reflects only Antarctic contributions^47^, whereas later growth periods may incorporate Northern Hemisphere ice as well. This ~9.6–9.3 Ma interval was also marked by enhanced North Atlantic poleward heat transport and a collapsed meridional temperature gradient, which reestablished before subsequent growth phases^5^. During this time, speleothem δ^18^O suggests hemispheric anti-phasing, with Antarctic ice expanding during intervals of elevated Arctic temperatures (and vice versa; Fig. 3). After ~8 Ma, following reestablishment of the North Atlantic temperature gradient^5^ and the onset of ephemeral Northern Hemisphere glaciations, a closer alignment between speleothem δ^18^O and global benthic δ^18^O (ref. ^49^) indicates that the two hemispheres operated more in-phase.

If the observed hemispheric anti-phasing and the inverse relationship between temperature and orbital forcing in the Arctic are accurate, they may have been partly driven by vegetation feedbacks, which played a key role in Late Miocene climate^1,19^. Over the past 12 Ma, Arctic forest canopy density peaked ~9.7 Ma, after which it became increasingly open, dominated by shrubs and herbs^50^. During the first speleothem growth phase, surface albedo was at its lowest, enhancing latent heat flux and atmospheric water vapour through increased evapotranspiration, contributing to elevated temperatures^19^. Despite weaker summer radiative forcing during low eccentricity–tilt phases, a longer warm season would have enabled positive vegetation feedbacks to persist over an extended period. As Arctic climate cooled through the Late Miocene, vegetation became more open^19^, the North Atlantic meridional temperature gradient increased^5^ and both Atlantic Meridional Overturning Circulation and North Atlantic Current strengthened^33^. This transition marked a shift towards a regime of transient glacial–interglacial cycles in the Arctic, operating in-phase with Antarctic ice sheet variability under shared orbital controls. High speleothem δ¹⁸O values generally coincided with high eccentricity–tilt and stronger summer seasonality, whereas low eccentricity–tilt facilitated sea-ice expansion.

The greatest sea-ice extent occurred during the final growth phase beginning ~5.57 Ma. During the preceding hiatus, the pronounced TG20 glacial occurred. The glacial cycles that followed were less extreme, and by interglacial TG15, Antarctic ice volume dropped sharply^38^. Glacial TG14 appears to have been a relatively minor glacial, and as it ended, speleothem deposition resumed despite elevated Na_ss_ levels indicating substantial sea-ice extent (Fig. 3). Sea ice may therefore have been present in the Arctic Ocean, even during the summer months, consistent with a moderately elevated CO_2_ scenario^20^. The sea ice remained, displaying high-amplitude high-frequency oscillations until ~5.53 Ma, then rapidly disappeared during the TG12 deglacial (Fig. 3). It expanded again for a short time during TG10, reaching peak expansion at the obliquity node. From ~5.49 to 5.38 Ma, Na_ss_ indicates persistently low sea ice with minor expansions, matching limited deep-sea δ^18^O variability^37,39,49^. At ~5.43 Ma, a rapid δ¹⁸O increase aligns with interglacial TG9^40^, paralleling rapid Antarctic deglaciation^38^.

Together, the δ¹⁸O and Na_ss_ time series reveal that eN.Greenland experienced pronounced Late Miocene climate and sea-ice variability. Clear connections to orbital forcing are difficult to reconcile within the limits of the chronology; however, it is clear that Late Miocene Arctic climate was much more dynamic and transient than has been captured thus far by modelling studies that are often limited to specific time slices and CO_2_ boundary conditions.

## Methods

### Geographical divisions

In this study, geographical subdivisions as defined by the Geological Survey of Denmark and Greenland Ministry of Energy, Utilities and Climate (GEUS) are used^51^.

### Geological setting

Solution caves in Ordovician and Silurian partly dolomitized limestones are widespread across North Greenland, having formed as large phreatic systems beneath an uplifted coastal peneplain ~1,000 m asl. Apatite fission track analysis dates this uplift to ~10 Ma, coinciding with the older of two Miocene uplift phases in North-East Greenland and southern areas^43,52^. Speleogenesis occurred concurrently with uplift, and in some locations, including the cave studied here, a small amount of vadose modification is evident as entrenchment and speleothem deposition^23,53^. However, the lack of vadose modification or extensive speleothem deposits indicates that most caves did not transmit large volumes of water following phreatic drainage, thereby supporting the existence of cold-based ice sheets during Quaternary glaciations^54^.

### Cave sampling location

Flowstone samples (KC19-7, KC19-9, KC19-12 and KC19-14) were collected from the rear of Cove Cave (Eqik Qaarusussuaq; KC Cave) ~100 m from the entrance where cave air temperature in July 2019 was recorded at −14.0 ± 0.6 °C using an Extech RH300 digital hygro-thermometer^23^. This spot cave air temperature measurement agrees within uncertainty with the MAAT from back trajectory analysis (−13.7 ± 1.0 °C). However, the contemporary cave morphology should not be considered as representative of when the analysed speleothems formed.

The sampling location contains a narrow vadose canyon, ~0.5 m wide and ~5 m deep (Extended Data Fig. 2). In situ flowstone drapes the palaeo-phreatic section and extends into the vadose canyon, indicating that vadose development predates speleothem deposition. Ex situ flowstone, presumed to have been broken through freeze–thaw processes, was sampled. KC19-7 was sampled from the extreme rear of the cave, at the top of the flowstone deposit, close to the roof. KC19-14 is a large broken curtain sequence sampled from the base of the flowstone sequence at the rear of the cave. Its original location could not be identified. It was cored due to its size. Before the vadose canyon, a boulder pile of broken speleothem was sampled ~15 m from KC19-7 and KC19-14. This pile yielded KC19-9 and KC19-12.

### Petrography and mineralogy

The speleothem fabrics were assessed using thin sections under transmitted-light and blue-light epifluorescence microscopy. To determine the mineralogical composition of the white layer, approximately 45 mg of powder was hand-drilled from the white layer of KC19-7. A small aliquot of the homogenized (by mortar and pestle) powder was measured by X-ray diffractometry using a High-Resolution Powder Bruker-AXS D8-Discover at the Institute of Mineralogy and Petrography, University of Innsbruck. Phase identification was achieved with DIFFRAC.SUITE software and the PDF4+ (2024) database. Calcite was found to be the only phase.

### Temperature reconstruction from reanalysis data

Greenland exhibits a large and abrupt temperature gradient from the continental interior to the coastline, particularly at the ice margin (Extended Data Fig. 3). Limited active monitoring near the study site inhibits precise characterization of local climate, because the nearest PROMICE weather stations (KPC-L and KPC-U) are located on the ice sheet, ~60–80 km southwest of KC Cave, and record a large temperature disparity (3.8 °C) over a short range (20 km). MAAT at KPC-L and KPC-U (from 2008 to 2023 CE) is −13.41 °C and −17.21 °C, respectively.

To estimate the 1991–2020 climatology at KC Cave, we obtained monthly 2-m temperatures from the fifth generation atmospheric reanalysis from the European Centre for Medium-Range Weather Forecasts (ERA-5)^55^, National Centers for Environmental Prediction and the National Center for Atmospheric Research (NCEP/NCAR)^56^, and the Copernicus Arctic Regional Reanalysis (CARRA)^57^ reanalysis datasets. Monthly means were calculated from CARRA’s 6-hourly data. For each dataset, MAAT was derived using a two-dimensional interpolant of a 3 × 3 grid centred on the grid cell containing the cave. Lapse-rate adjustments were applied for the elevation difference between model output and the cave site (660 m asl) but had a negligible effect (<0.8 °C).

Monthly reanalysis data from ERA-5 and NCEP/NCAR exhibit cold biases between 4 °C and 5 °C when applied to stations KPC-L and KPC-U. However, the 2-m MAAT in CARRA was within 0.09 °C and 0.16 °C, of observed values at KPC-L and KPC-U, respectively (Extended Data Fig. 3). Thus, we have high confidence in the CARRA-based climatology for KC Cave (Extended Data Fig. 3), which yields a MAAT of −13.73 °C (1991–2020) and a recent warming rate of 0.89 ± 0.13 °C per decade.

### Radiometric dating

Subsamples for U–Th dating were drilled in a thoroughly cleaned laminar flow hood used exclusively for low U concentration carbonate samples. Sample sizes ranged from 150 to 450 mg. Standard chemistry procedures were performed at the University of Minnesota to extract and purify U and Th aliquots^58^. Samples were spiked with a dilute mixed ^229^Th–^233^U–^236^U tracer to correct for instrumental fractionation and calculate U and Th concentrations and ratios. Measurements were conducted on a Thermo-Finnigan Neptune multicollector inductively coupled plasma mass spectrometer^59^ and ages calculated offline.

Laser ablation multi-collector inductively coupled plasma mass spectrometric (LA-MC-ICPMS) U–Pb dating was performed on highly polished slabs using laser ablation connected to a Thermo-Finnigan Neptune XT at the Institute of Global Environmental Change, Xi’an Jiaotong University^60^. Samples were ultrasonically cleaned in deionized water to remove surface contamination. Samples and reference materials (RMs) were placed in an S155 cell (Laurin Technics 155, aerosol dispersion volume <1 cm^3^), and ablated using a 193 nm ArF LA system (RESOlution LR). Aerosols were directly introduced into the LA-MC-ICPMS. Signal optimization used NIST616 and NIST614 glass, with conditions held constant during each run.

General parameters were 1.5–2.5 J cm^−2^ at 100 μm spot size, 10 Hz for 25 s, with 5–15 preablation shots for surface cleaning. Every eight samples, NIST614 glass and carbonate RMs of known age^60–62^ were analysed to correct for instrument drift and matrix effects. Iolite software^63^ was used for background subtraction, downhole effect correction and calculation of isotope ratios, errors, correlation coefficients and Tera–Wasserburg plots^64^. U–Pb ages were calculated using IsoplotR^65^.

The U/Pb ratio correction and U content (not strictly quantitative) were based on carbonate RMs ASH15^61^ and SB19-2^60^. Initial disequilibrium corrections for ^231^Pa, ^230^Th and ^226^Ra were omitted due to negligible effects in these old samples. ^234^Ui correction was also omitted, as its impact on young samples was <0.1 Ma—within error margins, especially when maximum error from RMs and mean square weighted deviation (MSWD) was applied. Furthermore, ^234^Ui values are not reliably known. Final reported errors for age and (^207^Pb/^206^Pb)_0_ are two standard errors, including sample dispersion and RM uncertainty.

Age models were constructed using Bayesian modelling in Oxcal^66,67^.

### Stable isotope analysis

Stable isotope analysis was conducted at the University of Innsbruck. Speleothem samples for δ^18^O and δ^13^C analysis were micromilled at 250 µm resolution and analysed on a Thermo Fisher Scientific DeltaV isotope ratio mass spectrometer linked to GasBench II^68^. In addition, 20 bedrock limestone samples were analysed, yielding δ^18^O values of −8.2 ± 0.3‰ and δ^13^C results of −0.1 ± 0.1‰ (2*σ*). All results are reported relative to the NBS19 standard on the Vienna Peedee belemnite (VPDB) scale. Analytical precision was 0.08‰ for δ¹⁸O and 0.06‰ for δ¹³C (1*σ*)^68^.

### Construction of the stacked composite stable isotope record

All four samples (KC19-7, KC19-9, KC19-12 and KC19-14) display comparable stable-isotope signals that, when cross-checked with macroscopic features (for example, petrographically inferred growth hiatuses and shared detrital-rich white layers), allow construction of a composite record and time series (Extended Data Fig. 5). Apart from the lower section of KC19-14 (118–200.4 mm), principal trends and perturbations are replicated in two to four samples across all intervals, with the strongest correlation between sample pairs KC19-7 versus KC19-9 and KC19-12 versus KC19-14 (Extended Data Fig. 5a–d). As KC19-14 contains the longest record (200.4 mm), it was used as the master record to construct the composite.

To combine the five micromilled tracks on a common depth scale, we tuned pairs of stable-isotope series using intrasite correlation age modelling (iscam) software in MatLab^69^. Iscam parameters included 500 AR1 simulations (each optimized with 1,000 Monte Carlo runs) and 10,000 Monte Carlo simulations of linearly interpolated age–depth models, with ages allowed to vary according to a Gaussian distribution.

Before input, synthetic ‘age models’ were created by treating 13–18 distinct isotopic, petrographic or macroscopic features in each pair as coeval events. Each event was assigned an ‘age’ corresponding to its depth in the master record and a 2*σ* uncertainty of 0.5 mm—twice the sampling resolution. KC19-7 was combined with its duplicate track to produce the KC19-7 composite, which was then tuned to KC19-9. KC19-12 was independently tuned to KC19-14, and the resulting pairwise composites were merged to form the master KC19 Composite δ¹⁸O and δ¹³C time series (Extended Data Fig. 5f,h, black lines) on a unified depth scale from 0 to 207.05 mm.

Individual iscam runs produced correlation coefficients (*r*) exceeding 0.9, and the robustness of the final composite is supported by strong replication of stable-isotope and elemental patterns across the shared depth scale (Extended Data Fig. 5f–h).

### Major- and trace-element analysis by LA-ICP-MS

Analyses were performed in line-scan mode at the Institute of Geosciences, JGU Mainz, Germany, using an ESI NWR193 ArF excimer laser ablation system with a TwoVol2 cell (193 nm wavelength), coupled to an Agilent 7700x quadrupole ICP-MS. Surfaces were preablated before each scan to remove surface contamination. Line scans were conducted at 15 µm s^−1^ using a rectangular beam (130 × 50 μm; 150 × 50 μm for preablation). The laser operated at 10 Hz with energy ~3.5 J cm^−2^. Background intensities were recorded for 15 s. Monitored isotopes included ^7^Li, ^23^Na, ^24^Mg, ^25^Mg, ^27^Al, ^31^P, ^32^S, ^34^S, ^39^K, ^44^Ca, ^47^Ti, ^49^Ti, ^52^Cr, ^53^Cr, ^55^Mn, ^56^Fe, ^56^Fe, ^59^Co, ^60^Ni, ^63^Cu, ^66^Zn, ^75^As, ^85^Rb, ^88^Sr, ^89^Y, ^90^Zr, ^93^Nb, ^95^Mo, ^111^Cd, ^133^Cs, ^138^Ba, ^139^La, ^1^Ce, ^208^Pb, ^232^Th and ^238^U.

Element concentrations were calibrated using synthetic glass NIST SRM 610 and preferred values from the GeoReM database^70,71^. Quality control materials (QCMs)—USGS MACS-3, USGS BCR-2G and NIST SRM 612—monitored accuracy and precision. Signals were collected in time-resolved mode and processed with iolite4 software^63^.

⁴³Ca was used as the internal standard, assuming a Ca concentration of 390,000 μg g^−1^ for samples, and the corresponding GeoReM values for calibration materials and QCMs. Averaged element concentrations from repeated QCM measurements (*n* = 15) agreed within 10% of reference values^72^ (excluding ^32^S and ^57^Fe) and had a relative standard deviation (1RSD) <10 %, except for P and S. Anomalous peaks were filtered by flattening >3*σ* deviations from a locally weighted smoothing spline. Elemental data were then smoothed to match the stable-isotope sampling resolution using a 19-point locally estimated scatterplot smoothing (LOESS) before plotting and principal component analysis.

Trace element tracks relative to the stable isotope tracks are accurate to within 1 mm.

### Modern δ^18^O of meteoric precipitation

No station measuring precipitation isotopes exists near the field site. We therefore used data from the Global Network of Isotopes in Precipitation, specifically from Station Nord, Danmarkshavn, and Scoresby Sund on Greenland’s east coast. A latitudinal gradient was established between these sites, yielding an estimated value for the field site of −22.9‰ (mean) or −22.8‰ (weighted mean).

### Correction of δ^18^O for PCP

Prior calcite precipitation (PCP) along flow paths in the epikarst and cave system alters the residual karst-water composition of δ¹³C, δ¹⁸O and certain elements (Mg, Sr, Ba and U), enriching values in secondary calcite above those produced by carbonate dissolution between CO_2_-rich meteoric water and bedrock or soil^73,74^. Isolating the time-varying PCP enrichment, particularly for δ¹⁸O, is key to reconstruct proxy signals reflecting climatic and environmental change, rather than in-cave processes.

We contend that pervasive enrichment of proxy data by PCP is evident through much of the KC19 record by: (1) an exceptional range in Mg concentration from 206 to 5,113 ppm, with most values falling above 920 ppm (Extended Data Fig. 5g); (2) broad covariance of Mg with Sr, Ba and U; (3) a statistically significant, positive covariance between ln(Mg/Ca) and ln(Sr/Ca) when Mg exceeds ~920 ppm (a positive ‘Sinclair test’), and; (4) the observation that eigenvectors for δ^18^O, Mg, Sr, Ba and U are nearly orthogonal to the trace-element suite associated with weathering and colloidal transport (Extended Data Fig. 8).

To back-calculate the δ¹⁸O shift from PCP, we used the proxy system model^73^, which simulates Rayleigh enrichment of Mg/Ca and δ¹⁸O from assumed initial karst-water values until observed concentrations in speleothem calcite are matched. As this approach involves several simplifying assumptions, we caution that our PCP-corrected δ¹⁸O series (Extended Data Fig. 8) reflect the maximum plausible enrichment, that is, the lowest likely δ¹⁸O values of bicarbonate originally present in seepage water. First, initial [Mg²⁺] in karst water was calculated from the lowest observed speleothem value using a temperature-dependent partition coefficient. This assumes constant ground temperature and that minimum Mg/Ca represents PCP-free conditions. Second, the Mg partition coefficient is assumed independent of groundwater [Mg²⁺], although experiments show it decreases at higher concentrations^74^. Third, aqueous [Ca²⁺] was estimated from site-specific controls on carbonate solubility, including temperature and soil *p*CO_2_. Finally, we assume PCP alone caused Mg enrichment above the minimum. Although LA-ICP-MS Mg data were available only for KC19-7 and KC19-14, the high intersample consistency in both elemental and stable-isotope data supports extending the Mg/Ca signal to KC19-9 and KC19-12 on the shared depth scale.

To assess uncertainty in assumed initial conditions, we performed sensitivity tests by varying model parameters across a plausible range for the Miocene cave environment (Extended Data Fig. 8). Initial [Ca²⁺] had the greatest influence on δ¹⁸O enrichment, as it directly affects calcite saturation and growth rate, modulating the extent of prior precipitation. Our median value of 1.84 mmol l^−1^ corresponds to equilibrium calcite dissolution at ~5,000 ppm CO_2_ in the soil–karst zone, comparable to modern mid- to high-latitude Eurasia^75^. End-member values of 1.28 and 2.45 mmol l^−1^ reflect ~1,500 and 15,000 ppm CO_2_, respectively, unlikely but within observed ranges for Arctic/sub-Arctic biomes. However, at [Ca²⁺] <1.84 mmol l^−1^, the model simulated unrealistically long PCP durations (>10,000 s) for high-Mg intervals, suggesting higher Ca^2+^ levels characterized the Miocene karst. The median cave temperature of 6 °C is consistent with Miocene warming, while end-member values of 1–15 °C span the plausible range. Water film thickness was varied from 5 to 15 μm. These variations in temperature and film thickness had relatively minor effects on PCP-corrected δ^18^O.

The sensitivity test involved calculating PCP-corrected δ^18^O series for all four samples using seven distinct parameter sets (Extended Data Fig. 8). These were merged on a common depth scale, and a 0.6-mm moving filter was applied to generate a kernel density function at each sampling depth. Although uncertainty reaches 3–4‰ in high-Mg intervals (for example, 48–106 mm depth), the main δ^18^O trends and perturbations remain robust—except under unrealistically low initial [Ca^2+^] scenarios (1 and 2). The final PCP-corrected δ^18^O composite (Extended Data Fig. 8e, black line) represents the median of the seven simulations across all samples and is interpreted as the primary meteoric water signal shaped by regional climatic variability. As incongruent calcite dissolution (ICD) can also cause similar cation covariance, we assessed it independently by examining temporal variance in Ba/Mg (Extended Data Fig. 9d). Dissolution experiments on the dolomitic limestone above Cove Cave (Eqik Qaarusussuaq) show ICD causes up to ~10-fold Ba enrichment relative to Mg and Sr when dissolution is <1%, while Sr/Mg remains largely unchanged. We infer ICD is minimal or absent during high-Mg intervals, which reflect strong PCP enrichment, and is highest during low-Mg, high-δ^18^O intervals—interpreted as the warmest and most humid periods.

### Correction of δ^13^C for PCP

Enrichment of δ^13^C from PCP along flow paths is typically greater than that of δ^18^O due to CO_2_ outgassing during calcite precipitation^74^. However, the δ^13^C enrichment factor is more difficult to constrain due to greater sensitivity to initial conditions and high natural variability in the slope of δ¹³C versus the fraction of residual [Ca^2+^] (fCa). Given these uncertainties, we did not construct a PCP-corrected δ^13^C composite like for oxygen. Instead, we use the proxy system model by ref. ^74^ to assess the potential influence of PCP on δ^13^C in the KC19 record. This model similarly assumes that the lowest Mg/Ca value reflects PCP-free conditions and applies a range of attenuation factors to the Mg partition coefficient to simulate reduced Mg uptake at elevated [Mg^2+^]. A mid-range slope of −8‰ for δ^13^C versus ln(fCa) was used to evaluate the potential δ^13^C effects.

In sample KC19-14, δ^13^C ranges from −6.2‰ (5th percentile) to −1.8‰ (95th percentile), while Mg/Ca is generally anticorrelated, varying from 1.89 to 14.56 mmol mol^−1^ across the same percentiles. This Mg/Ca range suggests fCa values of 0.12–0.54 in the highest-Mg, lowest-δ^13^C interval (~100–106 mm depth), yielding estimated initial (PCP-corrected) δ¹³C values of −22.9‰ to −11.2‰. In the lowermost section (>165 mm depth), where δ¹³C exceeds −4‰, fCa ranges from 0.15 to 0.95, giving initial δ¹³C values of −19.4‰ to −2.2‰. Regardless of model assumptions, correcting for PCP amplifies the observed δ¹³C trends, and thus does not alter the broader interpretations of this study.

### Orbital parameters

The precision of astronomical calculations of Earth’s orbital motion is considered reliable over the last 50 Ma (ref. ^48^). For the interval 9.6–9.0 Ma, obliquity phase uncertainty is estimated at ±1.6 kyr (ref. ^76^). As in previous studies, eccentricity and obliquity have been combined to construct an eccentricity–tilt composite^47,77^.

### Northern North Atlantic SSTs

Although the text describes nNA SSTs ‘relative to modern’^5,18^, ‘modern’ as defined here has some nuances due to a lack of preindustrial calibration. ‘Modern’ therefore refers to a multidecade average from ocean atlases.

## Online content

Any methods, additional references, Nature Portfolio reporting summaries, source data, extended data, supplementary information, acknowledgements, peer review information; details of author contributions and competing interests; and statements of data and code availability are available at 10.1038/s41561-025-01822-0.

## Supplementary information

Supplementary Information Supplementary Discussions 1–5.

Supplementary Table 1 U–Pb data table.

## Data Availability

Time-series stable isotope and trace element data are available via the National Centers for Environmental Information National Oceanic and Atmospheric Adminsitration at 10.25921/z522-b765 (ref. ^78^). All other data are provided with the Article.
