# Supplementary material for: Late Miocene Arctic warmth and terrestrial climate recorded by North Greenland speleothems
Source: Nat Geosci. 2025 Oct 21;18(12):1252–8. doi: 10.1038/s41561-025-01822-0 (PMC12685739; doi:10.1038/s41561-025-01822-0)
Supplement: Supplementary file 1 — Supplementary Discussions 1–5. [file 41561_2025_1822_MOESM1_ESM.pdf]

nature geoscience

Supplementary information

https://doi.org/10.1038/s41561-025-01822-0

Late Miocene Arctic warmth and terrestrial climate recorded by North Greenland speleothems

In the format provided by the authors and unedited

Contents

Supplementary Discussion 2
1. Speleothem Petrography and Hiatuses 2
2 Chronology 2
3 Insulated Ground Cover 3
4 Prior Calcite Precipitation 3
5 Na in Speleothems 3
References 4

Supplementary Discussion

1. Speleothem Petrography and Hiatuses

The speleothem fabric is predominantly elongated columnar calcite with individual crystals being a few mm on average, and occasionally up to 1-2 cm long (e.g., KC19-14). Columnar microcrystalline calcite is also present in some of the samples. All samples display regular fluorescent lamination (Extended Data (ED) Fig. 7b) under ultraviolet light. It is not known whether the lamination is annual in origin, however, considering the average lamina width of 5-20 µm, the thickness of the samples, and the period of deposition, it is not possible that growth was truly continuous.

None of the flowstones show signs of diagenetic alteration (e.g., mosaic calcite fabric). This is supported by the presence of regular, fine petrographic, and fluorescent lamination.

Macroscopic hiatuses are largely absent in the speleothems except for KC19-9, which displays an unconformity. Microscopically, two types of (micro-)hiatuses were observed in thin sections based on the presence/absence of: (i) dissolution features, (ii) unconformities, (iii) the lateral continuity of individual layers, (iv) the interruption of columnar calcite crystal growth, and; (v) the interruption on regular fluorescent lamination (ED Fig. 7c-j). The first type of hiatus is rare and characterised by a combination of at least two of these criteria (ED Fig. 7c-d). It likely represents a significant interruption of speleothem deposition. In contrast, the second type is abundant in all samples and is typically characterised by 25-50 µm thin "dust lines" rich in opaque inclusions associated with bright epifluorescence (ED Fig. 7c, h-j). These petrographic observations suggest that these micro-hiatuses represent comparably short gaps in calcite deposition.

The presence of three major hiatuses (in the KC samples) is indicated by the rapid change in growth rate in comparison to otherwise relatively constant growth rates across each of the main depositional periods (ED Fig. 6) and these correspond to microscopic (micro-)hiatuses, as described above.

The most prominent microscopic growth hiatus (hiatus-1 in the main text, see ED Fig.6) in the record is found at the base of the macroscopically distinctive white layer at 114-117 mm depth in KC19-14. We consider this microscopic hiatus, a type one (micro-)hiatus, whereby evidence for dissolution/corrosion and some recrystallisation is apparent in thin sections (ED Fig. 7e-g). The renewal of flowstone growth is indicated by an opaque, inclusion-rich micritic calcite layer, associated with bright epifluorescence, suggesting the presence of organic matter. Fluorescent lamination is difficult to resolve (ED Fig. 7g).

'KC-hiatus' and 'hiatus-2' are much less obvious than hiatus-1 in thin-section microscopy. 'KC-hiatus' (ED Fig. 7h-j) is only marked by a thin layer of "dust line" and a preceding approximately 1 mm thin zone lacking regular fluorescent lamination. The columnar calcite fabric seems unaffected by the growth interruption (ED Fig. 7e).

2 Chronology

The majority of samples dated with the U-Th method yield ages that are in secular equilibrium. Samples thus dated using the U-Pb method are also largely reliable with low concentrations of common Pb. The exception to this is in the top section of each sample (equivalent to c. 27.8 mm-from the top on the composite curve, ED Fig. 5) or the section younger than 5.38 Ma in the finished time-series (Fig. 2). In this upper section, finite U-Th ages for Quaternary glacial and interglacials were yielded by all samples (Supplementary Table 1). In addition, we were not able to obtain high precision U-Pb ages due to high concentrations of common Pb. Despite this, the U-Pb ages are clearly Late Miocene to early Pliocene in age and do not support the finite U-Th Quaternary ages. Given that the U-Th ages fall at the limit of the dating method, any minor disequilibrium could result in the calculation of a finite age, or alternatively a slight offset in spike calibration (e.g. due to evaporation) may also yield finite ages. Additionally, stable isotopes are consistent across the samples with no

major change between the lower dateable section and the upper chronologically challenging section; the petrography does not change either. Given these results, we consider the top 24 mm of the common record to likely be Late Miocene in age, rather than mid-Quaternary, as this would have implied a 4.5 Ma-long hiatus that is difficult to explain. Finally, it should be stated that the top 24 mm of the record is not a critical part of the presented and interpreted data.

We attempted to date the prominent white layer in KC19-7 and KC19-9, however, no reliable U-Pb ages could be obtained due to the high concentrations of common Pb.

Ages used in the final age model (n=32) include only those that did not yield high concentrations of common Pb (Supplementary Table 1) with the one exception being the age of 5.87 ± 0.58 Ma at 9.55 mm from the top, which is required to provide chronological control at the stratigraphically younger end of the record. Where the mean square of weighted deviates (MSWD)[85] is slightly elevated, the 2-sigma uncertainty corrected for overdispersion was applied.

3 Insulated Ground Cover

It is well observed that increased snow cover has the effect of insulating the ground and raising temperatures[86]. As an example, that would apply to the setting investigated here, based on modern analogues, a mean annual air temperature of ~ –5 °C combined with 25 % snow cover days, could have facilitated a mean annual ground temperature of 0 ºC[87].

4 Prior Calcite Precipitation

To further interpret Late Miocene climate variability in eastern North Greenland, we examine the speleothem $\delta^{18}O$ time series. The composite record shows values ranging from –13.6 to –17.3‰, comparable to Siberian Late Miocene speleothem $\delta^{18}O$ values of –14.6 to -17.5 ‰[(27)] (Fig. 2). Assuming calcite precipitation occurred near equilibrium at 0–11 °C, these values correspond to karst water $\delta^{18}O$ of –15.2 to –21.5‰$_{VSMOW}$. This range is consistent with $\delta^{18}O$ values of modern congelation cave ice and hoar frost in the region but is 1 to 8‰ heavier than modern meteoric precipitation (–22.8‰). This offset may be overestimated, as kinetic fractionation and prior calcite precipitation (PCP) can enrich speleothem $\delta^{18}O$[(31)]. However, the strong inter-sample reproducibility of $\delta^{18}O$ and $\delta^{13}C$ (ED Fig. 5) suggests minimal kinetic effects, consistent with a mild, slow-growth environment.

Throughout the record, Mg concentrations vary widely (206–5113 ppm) and positively correlate with Sr and Ba (ED Fig. 8), a pattern not fully explained by the incongruent dissolution of the partly dolomitic bedrock. Instead, it suggests systematic PCP-driven enrichment[74]. To isolate the climate signal, we estimated the time-varying $\delta^{18}O$ enrichment due to PCP[31] and applied a correction. A sensitivity analysis assessed the potential impact of PCP on the $\delta^{18}O$ record (ED Fig. 10). The resulting median, shown as the PCP-corrected composite time series (Fig. 2; ED Fig. 10), features a more negative $\delta^{18}O$ range of –15.8 to –21.9‰, corresponding to meteoric water values of –17.5 to –26‰$_{VSMOW}$.

5 Na in Speleothems

Speleothem Na is derived from environmental salinities associated with atmospheric precipitation (e.g., dust and sea spray) and weathering of the soil and epikarst, but due to its specific incorporation to interstitial positions within the calcite matrix, its concentration in the mineral (relative to cave waters) is highly sensitive to and positively correlated with growth-rate changes.

To test whether growth-rate changes constitute a dominant control on speleothem Na, we compare the Na signal to Sr/Mg, which has been shown to follow first-order changes in speleothem growth rate. This relationship (Sr/Mg) is due to the common inclusion of Sr in crystal lattice defects, leading to a higher partition coefficient (D_{Sr}) at higher growth rate, while Mg is relatively insensitive to this factor[74]. However, Na and Sr/Mg are uncorrelated throughout the entire KC19-7 and KC19-14 records,

including where Na is highly enriched, so speleothem Na variability must primarily be a function of environmental abundance.

Additionally, a co-plot of the natural logarithm of Na/Ca and Mg/Ca yields no correlation or slope, as might be expected if Na and Mg are derived from a common source and co-varied as a function of their respective partition coefficients. This observation is consistent with the orthogonal relationship between PCA eigenvectors of Na vs. Mg, Sr, Ba, and U (ED Fig. 8b). Thus, we conclude that Na variability in KC19 speleothems must derive from changes in extraneous Na sources, such as silicate weathering in the soil/epikarst and the sea-spray component of wet-atmospheric deposition.

Finally, to isolate atmospheric from silicate-weathering sources, we normalise speleothem Na to the detrital tracer of (Ti + Al) to derive Na_{SS}. We contend that this signal robustly reflects changes in the component of Na from marine aerosol deposition at the cave site, which may largely be a function of regional sea-ice extent.

References

[31]Skiba, V., Jouvet, G., Marwan, N., Spötl, C. & Fohlmeister, J. Speleothem growth and stable carbon isotopes as proxies of the presence and thermodynamical state of glaciers compared to modelled glacier evolution in the Alps. *Quaternary Science Reviews* **322**, 108403 (2023).

[74]Stoll, M. H. et al. Distinguishing the combined vegetation and soil component of $\delta^{13}C$ variation in speleothem records from subsequent degassing and prior calcite precipitation effects. *Climate of the Past* **19**, 2423-2444 (2023).

[85]Ludwig, K.R. & Titterington, D.M. Calculation of ^{230}ThU isochrons, ages, and errors. *Geochimica et Cosmochimica Acta* **58**, 5031-5042 (1994).

[86]Peng, X. *et al.* The thermal effect of snow cover on ground surface temperature in the Northern Hemisphere. *Environmental Research Letters* **19**, 044015 (2024).

[87]Töchterle, P., et al. Reconstructing Younger Dryas ground temperature and snow thickness from cave deposits. *Climate of the Past* **20**, 1521–1535 (2024).

[88]Cheng, H. *et al.* Improvements in ^{230}Th dating, ^{230}Th and ^{234}U half-life values, and U-Th isotopic measurements by multi-collector inductively coupled plasma mass spectrometry. *Earth and Planetary Science Letters* **371-372**, 82-91 (2013).

[89]Jaffey, A.H. *et al.* Precision measurement of half-lives and specific activities of ^{235}U and ^{238}U. *Physical Reviews C* **4**, 1889 (1971).

[90]Wedepohl, K.H. The Composition of the Continental Crust. *Geochimica et Cosmochimica Acta* 59, 1217-1232 (1995).
